# Independent multimerization of Latent TGFβ Binding Protein-1 stabilized by cross-linking and enhanced by heparan sulfate

**DOI:** 10.1038/srep34347

**Published:** 2016-09-28

**Authors:** Helen Troilo, Ruth Steer, Richard F. Collins, Cay M. Kielty, Clair Baldock

**Affiliations:** 1 The Wellcome Trust Centre for Cell-Matrix Research is within the School of Biological Sciences, Faculty of Biology, Medicine and Health, University of Manchester, Manchester, M13 9PT, UK

## Abstract

TGFβ plays key roles in fibrosis and cancer progression, and latency is conferred by covalent linkage to latent TGFβ binding proteins (LTBPs). LTBP1 is essential for TGFβ folding, secretion, matrix localization and activation but little is known about its structure due to its inherent size and flexibility. Here we show that LTBP1 adopts an extended conformation with stable matrix-binding N-terminus, extended central array of 11 calcium-binding EGF domains and flexible TGFβ-binding C-terminus. Moreover we demonstrate that LTBP1 forms short filament-like structures independent of other matrix components. The termini bind to each other to facilitate linear extension of the filament, while the N-terminal region can serve as a branch-point. Multimerization is enhanced in the presence of heparin and stabilized by the matrix cross-linking enzyme transglutaminase-2. These assemblies will extend the span of LTBP1 to potentially allow simultaneous N-terminal matrix and C-terminal fibrillin interactions providing tethering for TGFβ activation by mechanical force.

TGFβ is a potent cytokine with key roles in development and homeostasis. TGFβ is secreted in a latent form, non-covalently bound to its cleaved propeptide which is disulphide linked to LTBP family proteins which assist in folding and secretion^1^. However, there are also instances where LTBPs are secreted independent of TGFβ^2^. The TGFβ-binding LTBPs −1, −3 and −4 are critical for correct deposition and subsequent bioavailability of TGFβ in the extracellular matrix^3^. LTBP2 does not bind TGFβ and has TGFβ-independent functions^4^, while the other LTBPs are also involved in cell adhesion^5^, elastogenesis^6^ and stabilization of cell surface receptors^7^, independent of their TGFβ-binding role.

In addition to these roles, it has also been speculated that LTBPs may contribute to matrix architecture based on their similarity to the fibrillin family^8^. They are composed of three 8-cysteine domains, sixteen EGF-like repeats and a hybrid domain with proline-rich interdomain regions^9^. LTBP1 is known to be calcium binding and this interaction is thought to induce structural changes and protect the protein from proteolysis^10^. In TGFβ-binding members of the family, the latent complex associates with the second 8-cysteine domain^11^. In addition to localization of the growth factor, this association allows mechanical activation of TGFβ^12^ through interaction of the TGFβ prodomain with integrin α_V_β_6_^3^. Tensile force exerted through matrix-anchored LTBP1 and latent complex bound integrins alters the prodomain conformation releasing the growth factor^13^. The C-terminus of LTBP1 also associates with fibrillin^8^, while the N-terminus binds to heparin^14^,^15^, fibulin-5^16^ and fibronectin^17^. The N-terminus has been shown to be a substrate for transglutaminase-2 (TG2)^18^ which promotes its covalent incorporation into the extracellular matrix^19^. LTBPs can undergo alternative splicing as described for LTBP1^20^ (of which the best characterized are short-form (LTBP1S) and long-form^21^), LTBP2 and LTBP4^22^.

For a protein with clear mechanical functions, study of the structure is critical to a full understanding of its effects. The TGFβ binding domain has been previously solved revealing a disulfide bond which can be removed without disrupting domain fold, flanked by charged residues thought to mediate complex formation^23^. Moreover NMR studies have demonstrated that the domain pairs cbEGF-TB3 and TB3-EGF (both towards the C-terminal end of LTBP1) had high flexibility^24^ which may increase its accessibility for matrix interaction. The central region is occupied by eleven calcium-binding EGF-like (cbEGF) repeats. This type of domain arrangement has been predicted to produce a rod-like structure stabilized by calcium binding between neighboring domains^25^. However, in the related protein fibrillin-1, it produces an extended non-linear conformation^26^. A hinge region has been postulated to exist in the N-terminus of LTBP1 based on accessibility of a protease sensitive sequence^27^ though it is not clear whether this is a flexible region or whether it adapts discrete conformations in the presence of binding partners.

While it has been recently established that the function of LTBPs extends beyond TGFβ regulation^6^,^7^, the role of LTBPs in matrix architecture remains unresolved^8^. Here we investigate the nanostructure of LTBP1 and its capacity to assemble higher order oligomers in the absence of other factors. Our study demonstrates that the N-terminal region is rigid while the central cbEGF region is flexible but held in a predominantly extended conformation by calcium. We show calcium dependent multimerization of LTBP1 into filament-like structures which is N-terminally driven, promoted in the presence of heparin and stabilized by the matrix cross-linking enzyme TG2.

## Results

### LTBP1 can oligomerize via the N-terminus in a calcium dependent manner

**Figure 1:**
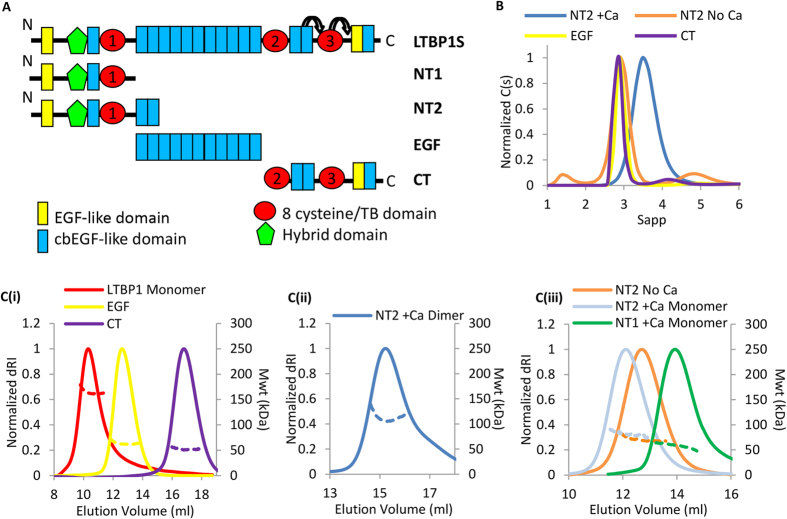
LTBP1 can oligomerize via the N-terminus in a calcium-dependent manner. (**A**) Schematic diagram showing the domain organization of full-length LTBP1S and the constructs NT1, NT2, EGF and CT. The three TB domains are shown as red circles and numbered and flexible regions either side of TB3 defined in ref. 24 are indicated by arrows. (**B**) AUC analysis of the constructs, the NT2 region is analyzed with either 2 mM calcium (blue) or 2 mM EDTA (orange) to show a monomer-dimer shift. (**C**) SEC-MALS analysis showing differential refractive index (dRI) solid line and molecular mass (kDa) dashed line as a function of elution volume for (i) LTBP1, EGF and CT, (ii) NT2 dimer in the presence of calcium, (iii) NT2 monomer in the presence of calcium or 2 mM EGTA and NT1 monomer in the presence of calcium. For panels C(i) and C(iii) a Superdex 200 column was used whereas for panel C(ii) a Superose 6 column was used.

Schematic diagrams of LTBP1S and the truncated constructs used in this study are shown in Fig. 1A. These were expressed in HEK-293 EBNA cells and purified (Fig. S1). These constructs were characterized using size exclusion chromatography (SEC)-multiangle light scattering (MALS) and analytical ultracentrifugation (AUC)(Fig. 1B,C, respectively) to determine oligomeric state and hydrodynamic properties (Table 1). While the EGF and CT constructs were found to be monomers, constructs containing the N-terminal domains also contained dimers and/or larger species in addition to the monomeric species in the presence of 2 mM CaCl_2_. The dimers and oligomers could be dissociated in the presence of 2 mM EDTA or EGTA (Fig. 1B,C and S1B) suggesting that the interaction is calcium dependent. Monomeric LTBP1, NT1 and NT2 species (Fig. 1C(i) and (iii)) could be separated from dimer and higher order oligomers by SEC but reassociated with time in the presence of calcium so were therefore used rapidly following purification for the analyses described below. The hydrodynamic data obtained from AUC, including the hydrodynamic radii and frictional ratios indicate that all the LTBP1 constructs are elongated and non-globular (Table 1).

### LTBP1 N-terminus has an elongated stable conformation

**Figure 2:**
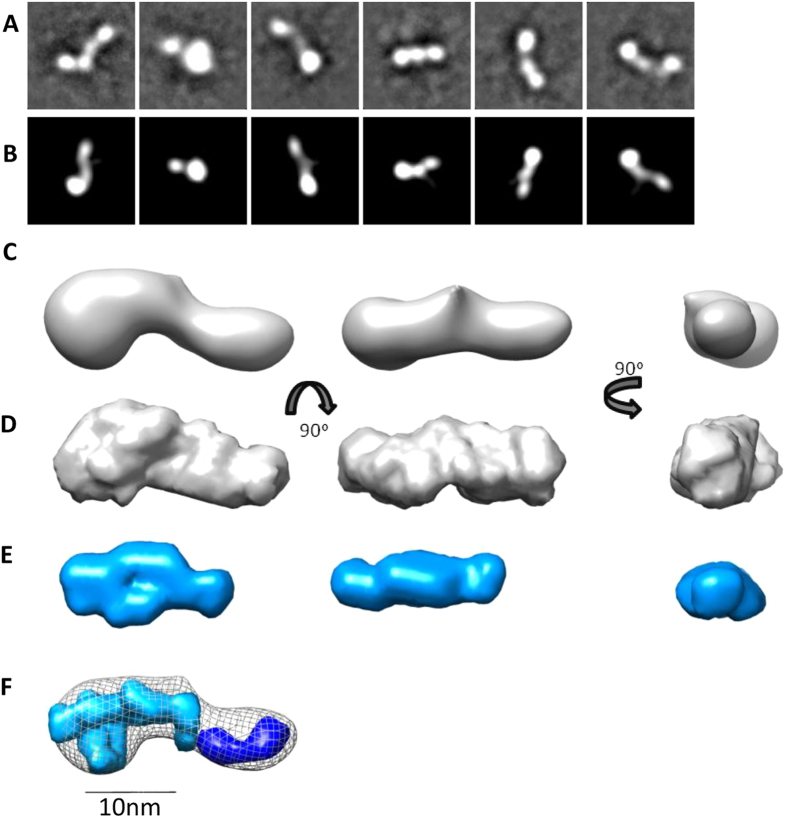
The N-terminal region of LTBP1 has an elongated stable conformation. (**A**). Negative staining EM of NT2, analyzing 10,000 particles from 150 micrographs. Representative class averages from 100 classes generated using EMAN2. (**B**) Matched projections from the 3D model (box size = 35.8 nm). (**C**) EM 3D reconstruction of NT2 shown in three orthogonal views. SAXS analysis of the NT2 and NT1 regions in solution. *Ab initio* models of NT2 (**D**) and NT1 (**E**) regions generated using DAMMIF software shown in three orthogonal views. (**F**) Modelling of the difference in density between the NT2 and NT1 regions was performed with MONSA. The NT1 density is shown in cyan and the additional density in the NT2 region is shown in blue and superimposed with the NT2 EM model. For panels (**C**–**F**) the scale bar = 10 nm.

The N-terminus of LTBP1 has many interdomain linkers and a putative hinge region^27^ so may be expected to demonstrate flexibility. To determine if this region was flexible, monomeric NT2 was prepared by SEC in the presence of calcium to isolate a monomeric species and was imaged by negative staining EM. 10,000 particles were selected and classified (Fig. 2A) and a 3D model at 24 Å resolution calculated by angular reconstitution (Fig. 2B,C). The convergence to a single model suggests that the protein is relatively inflexible. The structure had an elongated shape with dense lobe at one end, with dimensions 22 nm × 9 nm. The structure was further assessed in solution using small angle X-ray scattering (SAXS). Data on NT1 and NT2 regions both in the presence of calcium were obtained using SEC-SAXS, in which scattering measurements are made directly on the eluant from SEC to collect data on monomeric species. Data quality were assessed using the Guinier region (Figs S2 and S3) and to determine the radius of gyration (Rg) shown in Table 1. Analysis of flexibility from the Porod-Debye plot (Figs S2E and S3E) indicated that the N-terminal regions adopt a single conformation, a finding which supports the EM data. The maximum dimension (Dmax) for the NT2 region was 20 nm which is also consistent with the EM 3D model and 15 nm for the NT1 region. *Ab initio* models for the NT2 region strongly correlate with the EM model (Fig. 2D) and as expected the NT1 region was smaller with a shorter elongated protrusion (Fig. 2E). The relative densities of the NT1 and NT2 regions were modelled with MONSA^28^(Fig. 2F). The additional density present in the NT2 construct is consistent with the presence of an additional two cbEGF domains and locates the N-terminus in the larger lobe.

### LTBP1 N-terminus displays calcium dependent oligomerization enhanced by heparin binding

**Figure 3:**
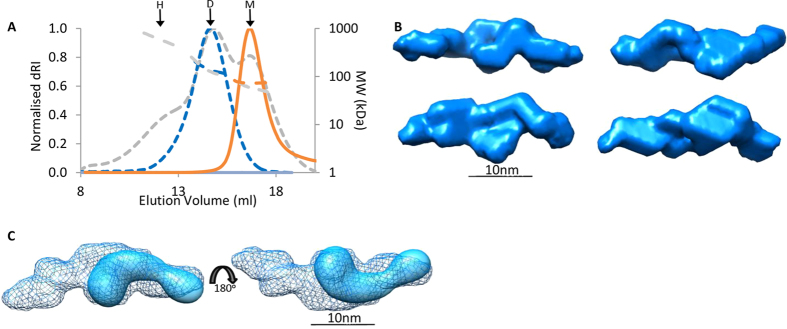
LTBP1 N-terminus multimerizes in a head-to-head manner. (**A**) SEC-MALS analysis of NT2 in 2 mM calcium or 2 mM EGTA on a Superose 6 column. Spontaneous oligomerization in the presence of calcium resulted in a mix of monomer (M = 54 kDa), dimer (D = 124 kDa) and higher order structures (H = 565 kDa) (gray trace) whereas in 2 mM EGTA only a monomer was observed (orange). To verify that the dominant species was a dimer, this fraction was run again (blue) and had Mwt of 135 kDa (Rh = 7.6 nm ± 1.017%). (**B**) SAXS data was collected on a dimer of the NT1 region and analyzed by ab initio modelling. A panel of four representative *ab initio* models is shown. (**C**) The NT1 monomer can be modelled into the dimer envelope suggesting an end-to-end arrangement.

We then investigated whether the N-terminal oligomers are able to form spontaneously in the absence of endogenous matrix components. From SEC, a monomer of NT2 was isolated and then stored overnight at 4 °C in either 2 mM calcium or 2 mM EGTA. These samples were then analyzed by MALS and in the absence of calcium (2 mM EGTA) the NT2 region remained monomeric. However, in the presence of calcium a mixture of distinct peaks corresponding approximately to monomer, dimer and higher order assemblies were observed (Fig. 3A). The mass measurement in MALS is more accurate for a single species therefore to verify the size of the predominant species, the dimer fraction was rerun immediately, confirming the dimer to be the major species. However, if the dimer was stored for longer periods of time, it would oligomerize into a mixture of dimer and higher order species. These data demonstrate that oligomerization can occur spontaneously *in vitro* in the absence of other factors to form an equilibrium of monomer, dimer and higher order oligomers and suggests that multimerization is calcium-dependent. To determine the arrangement of the N-terminal oligomers, SAXS data for a dimer of the NT1 region was collected. *Ab initio* models of the dimer were calculated in the absence of symmetry constraints and a panel of representative shapes shown (Fig. 3B). These all indicate a head-to-head arrangement via the globular region, suggesting an interaction mediated via the very N-terminal domains. This is further illustrated with the superimposition of the EM map which fits in this orientation into the dimer (Fig. 3C).

**Figure 4:**
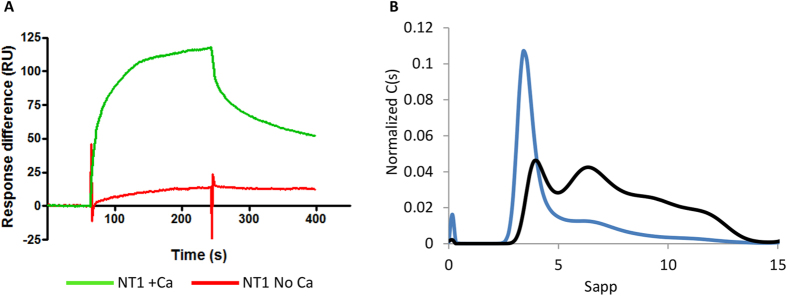
LTBP1 N-terminus displays calcium-dependent binding to heparin which promotes oligomerization. (**A**) Biacore analysis of 50 nM NT1 binding to immobilized heparin dp-20 in the presence of calcium (green) or 5 mM EGTA (red). (**B**) AUC analysis of NT1 in the absence (blue) and presence of heparin (black) showing increased propensity to form higher order oligomers.

Previous studies have shown that full length LTBP1 binds to heparin^29^. In order to investigate which region in LTBP1 is responsible for this interaction, Surface Plasmon Resonance was used to test for binding of each of the fragments against immobilized heparin dp-20 in the presence of calcium or EGTA. While no binding was observed to the cbEGF or C-terminal regions (Fig. S4), the N-terminus showed binding in a calcium-dependent manner (Fig. 4A). Heparin has previously been demonstrated to affect the oligomeric assembly of related matrix proteins^30^. We therefore investigated the larger structures formed by the N-terminus and whether heparin has an effect on multimerization. Whereas NT1 predominantly formed a dimer, when pre-incubated with heparin a range of higher order species were observed indicating heparin enhanced multimerization (Fig. 4B).

### The central cbEGF region of LTBP1 is held in an extended conformation by calcium

**Figure 5:**
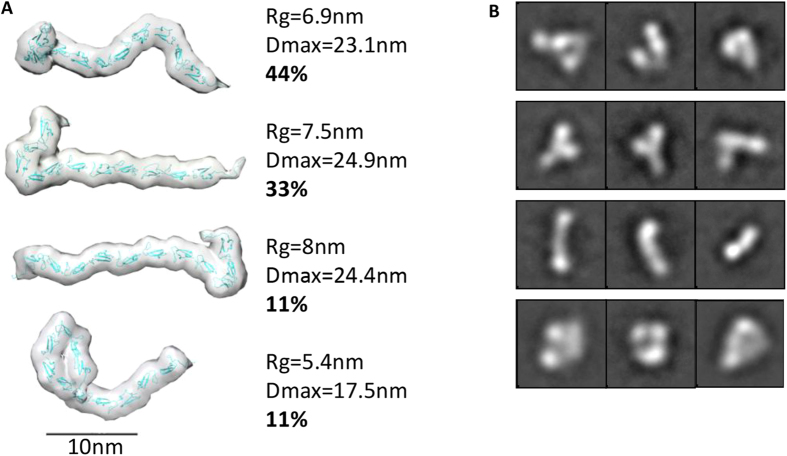
The central region of 11 cbEGF repeats of LTBP1 is flexible but held in an extended conformation by calcium. (**A**) Array of models produced using EOM from the EGF region X-ray scattering data in the presence of calcium. Extended conformations are more prevalent. (**B**) Class averages from negative stain EM showing a range of conformations reflected in the SAXS models, which are unable to resolve to a single 3D model (box size = 44.8 nm).

Arrays of cbEGF domains have been predicted to have a linear rod like arrangement based on the extrapolated structure of cbEGF domain pairs from fibrillin-1^25^. However, solution studies on longer arrays of cbEGF domains have indicated that they may have a more compact arrangement^26^. To determine how the 11 contiguous cbEGF domains in LTBP1 are structured, we used SAXS to determine their shape in solution. Data were collected in the presence and absence of calcium to determine how this effects their flexibility and conformation. In the presence of calcium this region had a Rg of 7.6 nm and an average Dmax of 23.2 nm, compared to a rod of 11 cbEGF domains which would have a theoretical Dmax of 32 nm and Rg of 9.6 nm. However after treatment with EDTA the Rg decreased to 5.4 nm indicating a conformational collapse on removal of calcium (Fig. S5). In the presence and absence of calcium the cbEGF region demonstrated flexibility as assessed by q^4^ plot^31^ (Fig. S5). Therefore, the data were processed assuming this region adopted an ensemble of different conformers in solution. Using the ensemble optimization method (EOM), 10,000 models were generated which are then compared with the experimental SAXS data to calculate an ensemble that together account for the scattering data. In the presence of calcium the ensemble contained four different conformations in different proportions with Rg ranging from 5.4–8 nm (Fig. 5A). The Rh values obtained from MALS and AUC and frictional ratio (Table 1) also suggest that elongated conformations are common in solution. These conformations ranged from an elongated structure with a kink at one end to a collapsed conformation with half the domains folding back on themselves. To validate whether these conformations are observed experimentally, the cbEGF region in the presence of calcium was imaged by EM. A range of class averages were observed that correlated with the models predicted indicating that this region has flexibility even in the presence of calcium (Fig. 5B). The overall conformation of this region is elongated but not linear and supports the concept that contiguous arrays of cbEGF domains are not rod-like units.

### The C-terminal LTBP1 region is flexible

**Figure 6:**
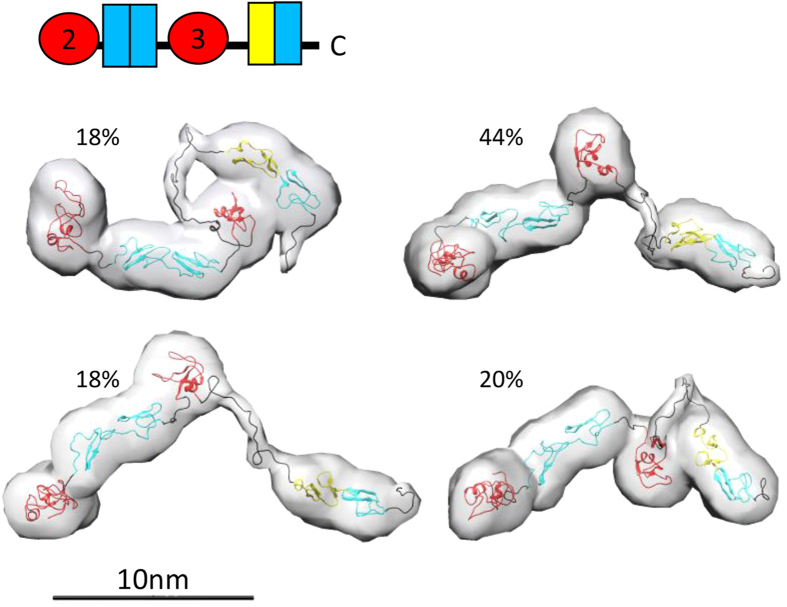
The C-terminal LTBP1 region is flexible. Array of models produced using the ensemble method from CT SAXS data with their relative proportion indicated. The two pairs of EGF domains in the CT construct were each treated as a rigid body. Scale bar = 10 nm.

Recent NMR studies have shown that the three terminal domains of LTBP1 have considerable domain-domain flexibility^24^. In order to determine if the C-terminal region of LTBP1 encompassing these domains plus the upstream domain responsible for binding latent TGFβ is also flexible we performed SAXS. These data indeed show that this longer region is flexible (Fig. S6) and ensemble analysis provides four models that collectively give rise to the scattering data. These models range in Rg from 4.5–6.2 nm and Dmax from 14.6–19.8 nm, which shows quite considerable variation in size, supporting the premise that this region is indeed flexible (Fig. 6). The Rg/Rh and frictional ratios are higher than expected for a globular protein suggesting moderate elongation of this region (Table 1).

### Assembly of LTBP1 via N-N or N-C terminal interactions

**Figure 7:**
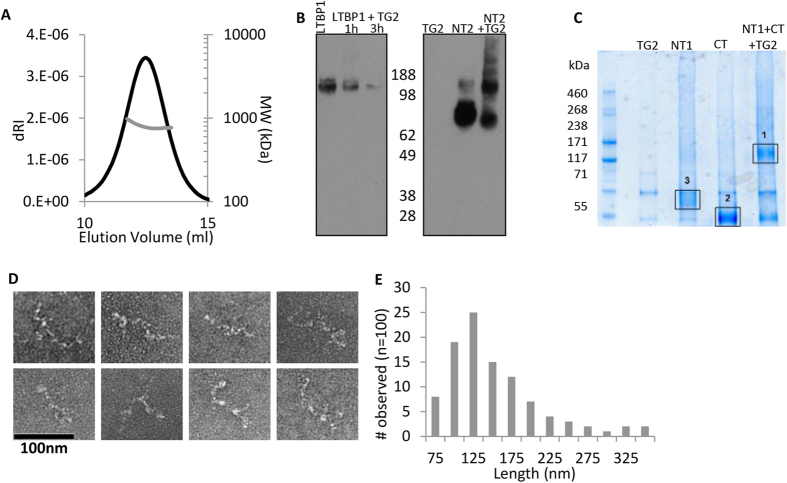
LTBP1 oligomerization via N-N or N-C terminal interactions. (**A**) SEC-MALS analysis of oligomeric LTBP1 indicates a molecular mass of 790 kDa. (**B**) Western blot showing cross-linking of LTBP1 using TG2, for full length LTBP1 a trace amount of dimer is visible after 1 hour. However after 3 hours the majority of cross-linked LTBP1 is too large to enter the gel. An equal amount of total protein was loaded into each well. The N-terminus alone forms monomer, dimer, trimer and tetramer with some higher order species. (**C**) TG2 cross-linking of the N- and C-terminal regions. Peptides from these bands were identified by MS with band 1 positive for both the N- and C-termini and bands 2 and 3 only positive for peptides from the C- or N-termini, respectively. (**D**) Oligomers were imaged using negative stain EM and analyzed using ImageJ. (**E**) Frequency of observation of the maximum straightened length of the oligomer (n = 100 from 32 images).

To determine the structure and oligomeric assembly of full-length LTBP1, it was purified and analyzed by MALS. Two main species were separated by SEC corresponding to a monomer and oligomer (Fig. S1B and Fig. 1C(i)). The molecular mass of the larger species was 790 kDa which is approximately 4–5 times the size of the monomer (Fig. 7A). In order to determine how this oligomer is formed, cross-linking experiments with TG2 were performed. It has already been demonstrated that LTBP1 is a substrate for TG2^18^ and that the N-terminal region is involved in cross-linking but it is thought that LTBP1 is cross-linked to fibronectin or other matrix components^32^. To determine whether LTBP1 was able to form inter-molecular transglutaminase cross-links, full-length LTBP1 was cross-linked by TG2 as evidenced by the disappearance of this species on SDS-PAGE gel (Fig. 7B), presumably as the cross-linked form became too large to enter the gel. The NT, EGF and CT regions were then incubated in the presence of TG2. Only the NT region was able to be cross-linked to itself (Fig. 7B and Fig. S7), providing further support for self-association at the N-terminus. However when the N- and C-terminal regions were incubated together higher molecular weight species formed containing peptides from both the N- and C-terminal regions indicating that these regions interact and this could be stabilized by a cross-link (Fig. 7C and Fig. S7). Together these data suggest that LTBP1 can self-assemble, in the absence of other matrix molecules, via N-N or N-C terminal interactions. To determine what form the higher order LTBP1 oligomers took, they were imaged by EM which showed thin elongated structures (Fig. 7D) with average length of 130 nm (Fig. 7E). These length measurements are consistent with 4–5 LTBP1 molecules in an end-to-end arrangement.

### LTBP1 is elongated with flexible regions

**Figure 8:**
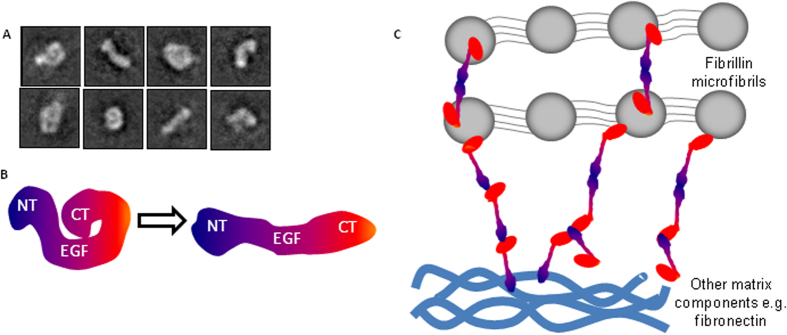
LTBP1 has flexible regions and can form small filamentous structures. (**A**) Selected class averages of full length LTBP1. Box size = 39.7 nm. (**B**) Cartoon schematic showing LTBP1 alternating between a compact and elongated conformation with flexibility toward the C-terminus. (**C**) Schematic diagram showing hypothesized arrangement of LTBP1 in the matrix with oligomers forming via N-N or N-C interactions cross-linked by TG2, with the N-terminus capable of acting as a branching point. In this way LTBP oligomers could span short distances between fibrillin microfibrils, and link fibrillin and fibronectin filaments.

In order to determine how the shorter constructs are arranged in the full-length molecule, monomeric full length LTBP1 was applied to grids for EM analysis (Fig. 8A). A range of classes were observed including elongated shapes and others more compact with the termini folding inwards. The maximum contour length (55 nm) of the compact particles was measured using ImageJ and approximately corresponds to the combined maximum dimensions of the NT1 (15 nm), EGF (23.2 nm) and CT regions (17.5 nm). Some particles appear to adopt more extended conformations, however other class averages are shorter than expected, presumably due to flexibility around the C-terminus. A model is therefore proposed in Fig. 8B, whereby LTBP1 has a rigid N-terminal region, an extendable EGF region and flexible C-terminus with the potential to either extend to give rise to elongated shapes or fold back to give rise to the compact conformations. The oligomers have regions of density consistent with both elongated and compact LTBP1 conformations and so it is probable that both forms exist within these assemblies. This model indicates an end-to-end arrangement consistent with the assembled oligomeric filaments. In summary, we propose in Fig. 8C the arrangement for full length LTBP1 deposition in the matrix whereby linear strands can form in an N-N or N-C orientation with the N-terminus also able to act as a branching point.

## Discussion

LTBP1 is essential for correct spatial and temporal regulation of TGFβ signaling and also important TGFβ-independent roles are emerging. However, to date studies on LTBP1 structure and assembly have been hampered by its large size and inherent flexibility^24^. Therefore we have determined the nanoscale structure of LTBP1 and higher order oligomers using combined structural and biochemical approaches. We show that the LTBP1 N-terminal region is not flexible, unlike the other regions of the molecule, and multimerizes into dimer and higher order oligomers in the presence of calcium. LTBP1 contains many calcium binding EGF domains and calcium levels in the matrix are typically many orders of magnitude higher than the intracellular concentration^33^. Our N-terminal constructs contain the putative “hinge” region, named because it contains protease-sensitive sequences flanked by ECM and TGFβ binding regions^27^, which suggests this region adopts a defined rather than flexible conformation perhaps able to adopt different conformations in the presence of binding partners. Full-length LTBP1 can adopt compact conformations with a flexible C-terminal region, these compact conformations may promote matrix diffusion and the partially buried C-terminus could serve to shield the growth factor from matrix proteases. Other conformations are more extended; the elongation of LTBP1 could contribute to mechanical force transduction from LTBP1 to the growth factor mediating integrin activation. Indeed tethering of the large latent TGFβ complex is required for force induced activation^12^.

It has been unclear whether LTBP1 is capable of forming its own fibrillar network or if it is dependent on scaffolds such as fibrillin or fibronectin for deposition^34^. LTBP1 and fibrillin have been shown to colocalize in early matrix deposition, yet in more mature cultures they occupy distinct networks^35^. Furthermore, fibroblasts lacking fibrillin1 did not deposit LTBP-1^36^ and knockdown of fibrillin1 also disrupted LTBP1 deposition^29^, however, tissues lacking fibrillin1 stained positively for LTBP1 suggesting other molecules such as fibronectin or fibrillin2 can compensate^37^. Our data show that LTBP1 is able to self-associate in an N- to N- and N- to C-terminal manner, supporting the concept that LTBP1 can autonomously assemble. Our data suggests that LTBP1 elongates in an end-to-end manner while the N-terminus may act as a branching point. Small filaments seen to bridge fibrillin microfibrils in the ciliary zonules of the eye^38^, could represent short LTBP filaments connecting adjacent microfibrils, indeed LTBP2 is required for correct ciliary zonule structure and microfibril bundling^4^.

LTBP1 can be cross-linked to other ECM components by the matrix cross-linking enzyme TG2^18^. We show that TG2 can also form cross-links between LTBP1 molecules, also confirming the specificity of self-association. The N-terminal region was able to cross-link to itself or to the C-terminus but no cross-links were detected between C-termini consistent with other studies^18^. In the absence of other matrix components, LTBP1 assembled preferentially into structures of approximately five monomer units as determined by mass and length. Interestingly, despite its similarity to fibrillin domain structure, LTBP1 forms elongated string-like structures whereas fibrillin forms bead-like globular structures^39^. However, LTBP1 and fibrillin also share structural features including an extended, non-linear conformation of cbEGF repeats^26^. While the independent LTBP1 structures are relatively short and without apparent bundling or periodicity, it is probable that LTBP1 assembly *in vivo* is heavily influenced by other matrix proteins.

Another candidate for influencing LTBP assembly is heparan sulfate (HS) and heparan sulfate proteoglycans (HSPGs). HS has previously been shown to bind to LTBP1^29^, and this interaction is conserved in LTBP2 which, like LTBP1, interacts via the N-terminus^15^. This binding may disrupt elastogenesis by displacing elastin from cell surface HSPGs^16^. However LTBP4 promotes elastogenesis^40^ suggesting that the effect of LTBPs on this pathway may be type and/or tissue specific. Our data reveal a novel feature of the LTBP-HS interaction, as in the presence of heparin (a more-sulfated variant of HS), LTBP1 has an increased capacity to self-assemble. The function of LTBP1 oligomerization is not yet known. However, loss of LTBP2 which is not directly involved in growth factor activation causes ciliary zonule fragmentation due to tissue specific failure of microfibril function^4^. This links to the emerging role for the LTBP family in stabilization of matrix components either by tethering to the matrix or by transmitting mechanical force^13^. This must function through the covalent tethering of the N-terminus of LTBPs to one protein and the C-terminus to another. One intriguing possibility is that oligomerization of LTBP1 extends its reach, allowing it to span longer distances between binding partners and may provide additional covalently-linked tethering sites to facilitate mechanical activation.

In summary, we show that LTBP1 has the capacity to form autonomous oligomeric structures in a calcium dependent manner which is promoted by heparin and interactions can be stabilized by TG2 cross-linking. The assembly is N-terminally driven and we propose a model of combined N- to C- and N- to N- elongation with the N-terminus also functioning as a branching point. Our structural data demonstrate that LTBP1 has the ability to self-assemble in the absence of other components and the capacity to directly contribute to matrix architecture.

## Materials and Methods

### Protein Expression and Purification

Constructs LTBP1S, CT (residues 1002–1395)^29^, NT1 (residues 21–488), NT2 (residues 21–630), and EGF region (residues 526–1014) were expressed in HEK-293 EBNA cells and purified using C-terminal polyhistidine tags via nickel affinity chromatography followed by size exclusion chromatography (SEC). Purity of the constructs was verified using SDS-PAGE and the identity of proteins confirmed using Mass Spectrometry (MS) of excised bands.

### Multi Angle Light Scattering

Samples (0.5 ml at ~0.5 mg ml^−1^) were injected onto a SEC column (Superdex 200 or Superose 6) at 0.75 ml min^−1^. Unless otherwise stated, buffer conditions were 10 mM Tris, 150 mM NaCl, 2 mM CaCl_2_, pH 7.4. Calcium concentrations reflect levels in the matrix and those used for previous studies on other members of the fibrillin superfamily^26^,^41^. Samples were passed through a Wyatt DAWN Heleod II EOS 18-angle laser photometer. This was coupled to a Wyatt Optilab rEX refractive index detector and data were analyzed using Astra 6 software.

### Analytical Ultracentrifugation

Samples (~0.5 mg ml^−1^) were analyzed in the same buffers as for MALS. Studies were carried out using a Beckman XL-A ultracentrifuge as described previously^42^. To study changes in the presence of heparin, samples were pre-incubated for 12 hours with an approximately 10x molar excess of low molecular weight heparin (Iduron, 1800–7500 Da). Unbound heparin was removed using SEC.

### Small Angle X-ray Scattering

Data were collected using inline SEC-SAXS on a Superdex 200 3.2/300 column at the ESRF on Beamline BM29 and at Diamond Light Source on beamline B21. Data were collected at 1 second intervals and the buffer blank was the eluent after one column volume. The scattering images obtained were spherically averaged using in-house software then protein scattering intensities were scaled and merged for each frame with a consistently similar Rg across the SEC elution peak and buffer subtracted using ScÅtter http://www.bioisis.net/tutorial/9. *Ab initio* modelling was performed using DAMMIF^43^ software in slow mode. The representative model had the lowest normalized spatial discrepancy from 20 repeats compared using DAMSEL^44^. Multiphase volumetric modelling was performed using MONSA^28^ to analyze the difference in density between NT1 and NT2 constructs. SwissModel was used to generate models of the individual domains with the exception of TB3 for which the structure is known^23^. As the EGF and CT regions were flexible they were analyzed as an ensemble, 10,000 models were generated using Ranch. Ranch generates 10,000 independent models using the amino acid sequence and high resolution structures of domains as the input. Within these constraints the models have random conformation and aim to cover all possible conformations. An ensemble of models representing the experimental SAXS data was generated by EOM from the pool of 10,000 models^45^.

### Negative Stain EM

Full length LTBP1S, EGF and NT2 were negatively stained as in ref. 42. Data were recorded at 23000 × on a Tecnai Biotwin at 120 kV with a Gatan Orius CCD camera with a 1 s exposure at 0.5–1.5 μm defocus range at 2.8 Å/pixel for NT2 and 3.5 Å/pixel for EGF. Data were recorded on a FEI G2 Polara at 300 keV for LTBP1S at 3.1 Å/pixel. Using EMAN2^46^, 10,000 particles were picked for EGF and NT2 and 2500 for full length LTBP1 using a combination of manual and semi-automated picking. Contrast function was corrected and each dataset subject to 2D classification. For NT2, these were used to generate an initial 3D model to seed six rounds of iterative refinement to produce a self-consistent 3D structure. Data was split into odd and even numbers and each refined independently to produce a resolution curve for each stage of refinement. With a Fourier shell correlation cut-off value of 0.143, the resolution estimate was 24 Å. Modelling was performed using UCSF Chimera^47^.

### Cross-linking

Guinea pig liver derived commercial transglutaminase-2 (Sigma-Aldrich) was incubated with LTBP1 fragments in a ratio of 0.1:1 for 2 hours at 30 °C in 10 mM Hepes, 150 mM NaCl pH 7.4 (HBS) buffer containing 1 mM CaCl_2_. When cross-linking mixed samples, each component was included at equal molar ratios. Reactions were halted by heating to 90 °C and addition of detergent. Products were analyzed by western blotting using anti-histidine monoclonal antibody (R&D Systems).

### Surface Plasmon Resonance

50 nM of recombinant fragments in HBS buffer with 0.005% (v/v) Tween-20 were injected over immobilized heparin saccharide dp-20 (Iduron) biotinylated using the cis-diol method on a streptavidin sensor chip (GE Healthcare). For calcium depleted conditions samples were prepared with 5mM of EGTA. All analyses were performed at least twice and traces were evaluated using BIAvaluation software (Biacore (GE Healthcare)).

## Additional Information

**How to cite this article**: Troilo, H. *et al.* Independent multimerization of Latent TGFβ Binding Protein-1 stabilized by cross-linking and enhanced by heparan sulfate. *Sci. Rep.* **6**, 34347; doi: 10.1038/srep34347 (2016).

## Supplementary Material

Supplementary Information

## Figures and Tables

**Table 1:** Hydrodynamic parameters from different biophysical techniques.

		NT2		EGF	CT
Hydrodynamic Properties		Monomer	Dimer		
Molecular Weight (kDa)	Sequence	66	132	54.5	45
	MALS	70.9*	135	62.5	52.5
	AUC	77*	186	72	52
Dmax (nm)	SAXS	20.0	N.D.	23.2	17.5
Rg (nm)	SAXS	6	N.D.	7.6	4.9
Rh (nm)	MALS	6.23*	7.9	7.27	5.26
	AUC	6*	8.2	6.64	5.51
f/f0	AUC	1.95*	2.44	2.87	2.34
S_20_W	AUC	3.08*	3.9	2.7	2.75

N.D. = not determined.

^*^Data collected in the presence of 2 mM EDTA.

## References

[b1] Miyazono K., Olofsson A., Colosetti P. & Heldin C. H. A role of the latent TGF-beta 1-binding protein in the assembly and secretion of TGF-beta 1. EMBO J 10, 1091–1101 (1991). 2022183 10.1002/j.1460-2075.1991.tb08049.x PMC452762

[b2] Kantola A. K., Ryynanen M. J., Lhota F., Keski-Oja J. & Koli K. Independent regulation of short and long forms of latent TGF-beta binding protein (LTBP)-4 in cultured fibroblasts and human tissues. J Cell Physiol. 223, 727–736 (2010). 20175115 10.1002/jcp.22082

[b3] Annes J. P., Chen Y., Munger J. S. & Rifkin D. B. Integrin alphaVbeta6-mediated activation of latent TGF-beta requires the latent TGF-beta binding protein-1. J Cell Biol. 165, 723–734 (2004). 15184403 10.1083/jcb.200312172 PMC2172370

[b4] Inoue T. *et al.* Latent TGF-beta binding protein-2 is essential for the development of ciliary zonule microfibrils. Hum Mol Genet 23, 5672–5682 (2014). 24908666 10.1093/hmg/ddu283 PMC4189902

[b5] Vehvilainen P., Hyytiainen M. & Keski-Oja J. Latent transforming growth factor-beta-binding protein 2 is an adhesion protein for melanoma cells. J Biol Chem. 278, 24705–24713 (2003). 12716902 10.1074/jbc.M212953200

[b6] Dabovic B. *et al.* Function of latent TGFbeta binding protein 4 and fibulin 5 in elastogenesis and lung development. J Cell Physiol. 230, 226–236 (2015). 24962333 10.1002/jcp.24704 PMC4436707

[b7] Su C. T. *et al.* Latent transforming growth factor binding protein 4 regulates transforming growth factor beta receptor stability. Hum Mol Genet 24, 4024–4036 (2015). 25882708 10.1093/hmg/ddv139 PMC4476448

[b8] Isogai Z. *et al.* Latent transforming growth factor beta-binding protein 1 interacts with fibrillin and is a microfibril-associated protein.J Biol Chem. 278, 2750–2757 (2003). 12429738 10.1074/jbc.M209256200

[b9] Oklu R. & Hesketh R. The latent transforming growth factor beta binding protein (LTBP) family. Biochem J 352 **Pt 3**, 601–610 (2000). 11104663 PMC1221494

[b10] Colosetti P., Hellman U., Heldin C. H. & Miyazono K. Ca2+ binding of latent transforming growth factor-beta 1 binding protein.FEBS Lett. 320, 140–144 (1993). 8458430 10.1016/0014-5793(93)80079-a

[b11] Gleizes P. E., Beavis R. C., Mazzieri R., Shen B. & Rifkin D. B. Identification and characterization of an eight-cysteine repeat of the latent transforming growth factor-beta binding protein-1 that mediates bonding to the latent transforming growth factor-beta1. J Biol Chem. 271, 29891–29896 (1996). 8939931 10.1074/jbc.271.47.29891

[b12] Buscemi L. *et al.* The single-molecule mechanics of the latent TGF-beta1 complex. Curr Biol 21, 2046–2054 (2011). 22169532 10.1016/j.cub.2011.11.037

[b13] Shi M. *et al.* Latent TGF-beta structure and activation. Nature 474, 343–349 (2011). 21677751 10.1038/nature10152 PMC4717672

[b14] Chen Q. *et al.* Potential role for heparan sulfate proteoglycans in regulation of transforming growth factor-beta (TGF-beta) by modulating assembly of latent TGF-beta-binding protein-1. J Biol Chem. 282, 26418–26430 (2007). 17580303 10.1074/jbc.M703341200

[b15] Parsi M. K., Adams J. R., Whitelock J. & Gibson M. A. LTBP-2 has multiple heparin/heparan sulfate binding sites. Matrix Biol. 29, 393–401 (2010). 20382221 10.1016/j.matbio.2010.03.005

[b16] Sideek M. A., Menz C., Parsi M. K. & Gibson M. A. LTBP-2 competes with tropoelastin for binding to fibulin-5 and heparin, and is a negative modulator of elastinogenesis. Matrix Biol. 34, 114–123 (2014). 24148803 10.1016/j.matbio.2013.10.007

[b17] Kantola A. K., Keski-Oja J. & Koli K. Fibronectin and heparin binding domains of latent TGF-beta binding protein (LTBP)-4 mediate matrix targeting and cell adhesion. Exp Cell Res. 314, 2488–2500 (2008). 18585707 10.1016/j.yexcr.2008.05.010

[b18] Nunes I., Gleizes P. E., Metz C. N. & Rifkin D. B. Latent transforming growth factor-beta binding protein domains involved in activation and transglutaminase-dependent cross-linking of latent transforming growth factor-beta. J Cell Biol. 136, 1151–1163 (1997). 9060478 10.1083/jcb.136.5.1151 PMC2132473

[b19] Verderio E. *et al.* Regulation of cell surface tissue transglutaminase: effects on matrix storage of latent transforming growth factor-beta binding protein-1. J Histochem Cytochem. 47, 1417–1432 (1999). 10544215 10.1177/002215549904701108

[b20] Oklu R., Hesketh T. R., Metcalfe J. C. & Kemp P. R. Expression of alternatively spliced human latent transforming growth factor beta binding protein-1. FEBS Lett. 435, 143–148 (1998). 9762896 10.1016/s0014-5793(98)01054-0

[b21] Olofsson A. *et al.* Efficient association of an amino-terminally extended form of human latent transforming growth factor-beta binding protein with the extracellular matrix.J Biol Chem. 270, 31294–31297 (1995). 8537398 10.1074/jbc.270.52.31294

[b22] Michel K. *et al.* Analysis of the expression pattern of the latent transforming growth factor beta binding protein isoforms in normal and diseased human liver reveals a new splice variant missing the proteinase-sensitive hinge region. Hepatology 27, 1592–1599 (1998). 9620332 10.1002/hep.510270619

[b23] Lack J. *et al.* Solution structure of the third TB domain from LTBP1 provides insight into assembly of the large latent complex that sequesters latent TGF-beta. J Mol Biol. 334, 281–291 (2003). 14607119 10.1016/j.jmb.2003.09.053

[b24] Robertson I. B., Handford P. A. & Redfield C. NMR spectroscopic and bioinformatic analyses of the LTBP1 C-terminus reveal a highly dynamic domain organisation. Plos One 9, e87125 (2014). 24489852 10.1371/journal.pone.0087125 PMC3906135

[b25] Downing A. K. *et al.* Solution structure of a pair of calcium-binding epidermal growth factor-like domains: implications for the Marfan syndrome and other genetic disorders. Cell 85, 597–605 (1996). 8653794 10.1016/s0092-8674(00)81259-3

[b26] Baldock C. *et al.* Nanostructure of fibrillin-1 reveals compact conformation of EGF arrays and mechanism for extensibility. Proc Natl Acad Sci USA 103, 11922–11927 (2006). 16880403 10.1073/pnas.0601609103 PMC1567674

[b27] Taipale J., Miyazono K., Heldin C. H. & Keski-Oja J. Latent transforming growth factor-beta 1 associates to fibroblast extracellular matrix via latent TGF-beta binding protein. J Cell Biol. 124, 171–181 (1994). 8294500 10.1083/jcb.124.1.171 PMC2119892

[b28] Svergun D. I. Restoring low resolution structure of biological macromolecules from solution scattering using simulated annealing. Biophys J. 76, 2879–2886 (1999). 10354416 10.1016/S0006-3495(99)77443-6 PMC1300260

[b29] Massam-Wu T. *et al.* Assembly of fibrillin microfibrils governs extracellular deposition of latent TGF beta. J Cell Sci. 123, 3006–3018 (2010). 20699357 10.1242/jcs.073437 PMC2923573

[b30] Tiedemann K., Batge B., Muller P. K. & Reinhardt D. P. Interactions of fibrillin-1 with heparin/heparan sulfate, implications for microfibrillar assembly. J Biol Chem. 276, 36035–36042 (2001). 11461921 10.1074/jbc.M104985200

[b31] Rambo R. P. & Tainer J. A. Characterizing flexible and intrinsically unstructured biological macromolecules by SAS using the Porod-Debye law. Biopolymers 95, 559–571 (2011). 21509745 10.1002/bip.21638 PMC3103662

[b32] Taipale J., Saharinen J., Hedman K. & Keski-Oja J. Latent transforming growth factor-beta 1 and its binding protein are components of extracellular matrix microfibrils. J Histochem Cytochem 44, 875–889 (1996). 8756760 10.1177/44.8.8756760

[b33] Koch A. W., Engel J. & Maurer P. Calcium Binding to Extracellular Matrix Proteins, Functional and Pathological Effects. The Molecular Basis of Calcium Action in Biology and Medicine 1, 151–164 (2000).

[b34] Unsold C., Hyytiainen M., Bruckner-Tuderman L. & Keski-Oja J. Latent TGF-beta binding protein LTBP-1 contains three potential extracellular matrix interacting domains. J Cell Sci. 114, 187–197 (2001). 11112702 10.1242/jcs.114.1.187

[b35] Dallas S. L. *et al.* Role of the latent transforming growth factor beta binding protein 1 in fibrillin-containing microfibrils in bone cells *in vitro* and *in vivo*. J Bone Miner Res. 15, 68–81 (2000). 10646116 10.1359/jbmr.2000.15.1.68

[b36] Ono R. N. *et al.* Latent transforming growth factor beta-binding proteins and fibulins compete for fibrillin-1 and exhibit exquisite specificities in binding sites. J Biol Chem. 284, 16872–16881 (2009). 19349279 10.1074/jbc.M809348200 PMC2719323

[b37] Zilberberg L. *et al.* Specificity of latent TGF-beta binding protein (LTBP) incorporation into matrix: role of fibrillins and fibronectin. J Cell Physiol. 227, 3828–3836 (2012). 22495824 10.1002/jcp.24094 PMC3404192

[b38] Davis E. C., Roth R. A., Heuser J. E. & Mecham R. P. Ultrastructural properties of ciliary zonule microfibrils. J Struct Biol. 139, 65–75 (2002). 12406689 10.1016/s1047-8477(02)00559-2

[b39] Hubmacher D. *et al.* Biogenesis of extracellular microfibrils: Multimerization of the fibrillin-1 C terminus into bead-like structures enables self-assembly. Proc Natl Acad Sci USA 105, 6548–6553 (2008). 18448684 10.1073/pnas.0706335105 PMC2373353

[b40] Noda K. *et al.* Latent TGF-beta binding protein 4 promotes elastic fiber assembly by interacting with fibulin-5. Proc Natl Acad Sci USA 110, 2852–2857 (2013). 23382201 10.1073/pnas.1215779110 PMC3581912

[b41] Rock M. J. *et al.* Molecular basis of elastic fiber formation. Critical interactions and a tropoelastin-fibrillin-1 cross-link. J Biol Chem. 279, 23748–23758 (2004). 15039439 10.1074/jbc.M400212200

[b42] Troilo H. *et al.* Nanoscale structure of the BMP antagonist chordin supports cooperative BMP binding. Proc Natl Acad Sci USA 111, 13063–13068 (2014). 25157165 10.1073/pnas.1404166111 PMC4246984

[b43] Franke D. & Svergun D. I. DAMMIF, a program for rapid ab-initio shape determination in small-angle scattering. J. Appl. Cryst. 42, 342–346 (2009). 27630371 10.1107/S0021889809000338 PMC5023043

[b44] Kozin M. & Svergun D. I. Automated matching of high- and low-resolution structural models. J. Appl. Cryst. 34, 33–41 (2001).

[b45] Bernado P., Mylonas E., Petoukhov M. V., Blackledge M. & Svergun D. I. Structural characterization of flexible proteins using small-angle X-ray scattering. J Am Chem Soc. 129, 5656–5664 (2007). 17411046 10.1021/ja069124n

[b46] Tang G. *et al.* EMAN2: an extensible image processing suite for electron microscopy. J Struct Biol. 157, 38–46 (2007). 16859925 10.1016/j.jsb.2006.05.009

[b47] Pettersen E. F. *et al.* UCSF Chimera--a visualization system for exploratory research and analysis. J Comput Chem. 25, 1605–1612 (2004). 15264254 10.1002/jcc.20084

