# Supplementary material for: Independent multimerization of Latent TGFβ Binding Protein-1 stabilized by cross-linking and enhanced by heparan sulfate
Source: Sci Rep. 2016 Sep 28;6:34347. doi: 10.1038/srep34347 (PMC5039643; doi:10.1038/srep34347)
Supplement: Supplementary Information [file srep34347-s1.pdf]

Independent multimerization of Latent TGFβ Binding Protein-1 stabilized by cross-linking and enhanced by heparan sulfate

Helen Troilo, Ruth Steer, Richard F Collins, Cay M Kielty and Clair Baldock.

Supplementary Figure 1

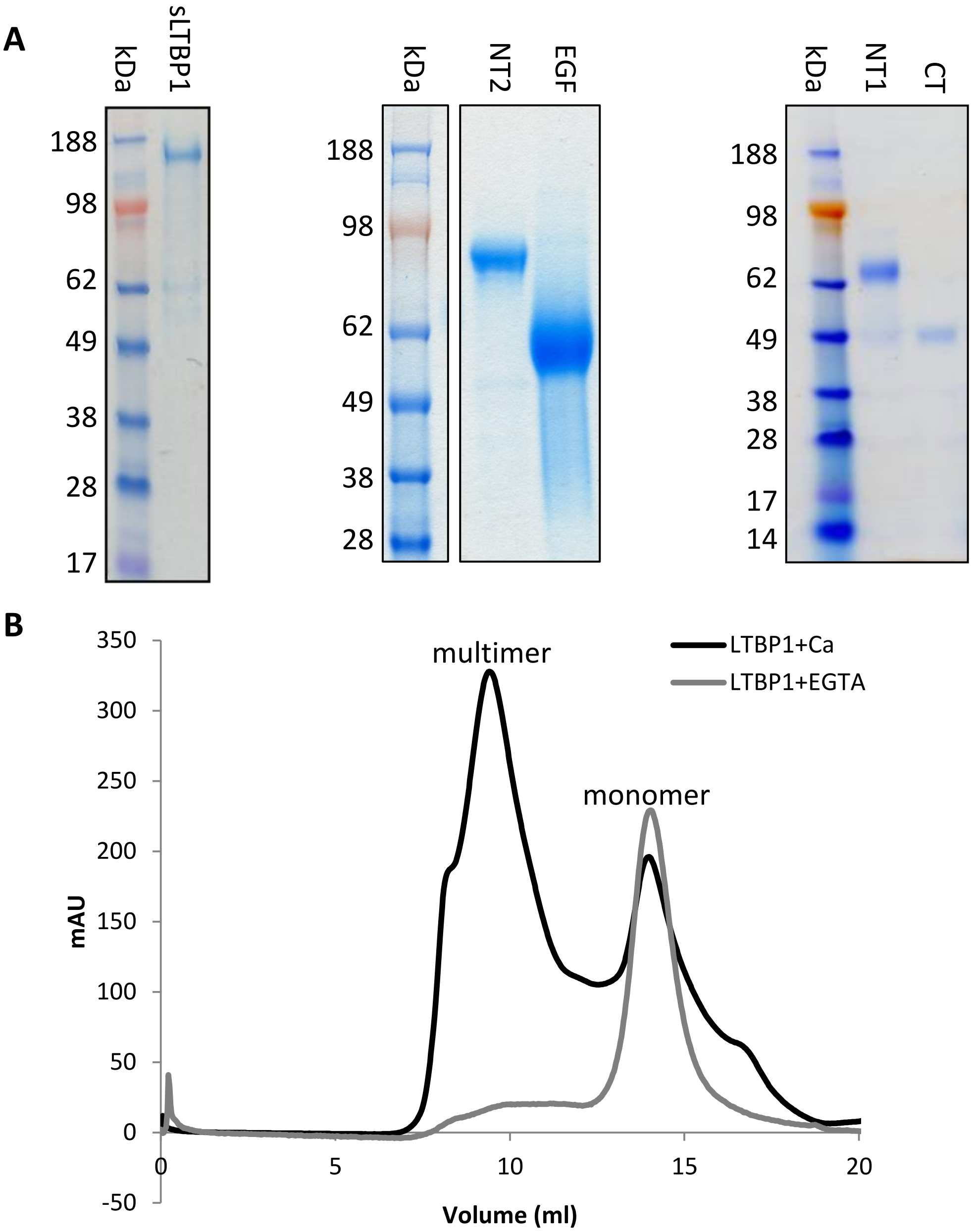

Supplementary Figure 1: Purification of LTBP1 constructs. (A) Non-reduced coomassie blue stained SDS-PAGE gels showing the purified constructs: LTBP-1S, NT1, NT2, EGF and CT. (B) Size exclusion chromatogram of LTBP1 in the presence of calcium showing multimer and monomer species (black trace). These species were further analysed by SEC-MALS in figures 1C(i) and 7A. In the absence of calcium (2mM EGTA), LTBP1 is predominantly a monomer (grey trace).

Supplementary Figure 2

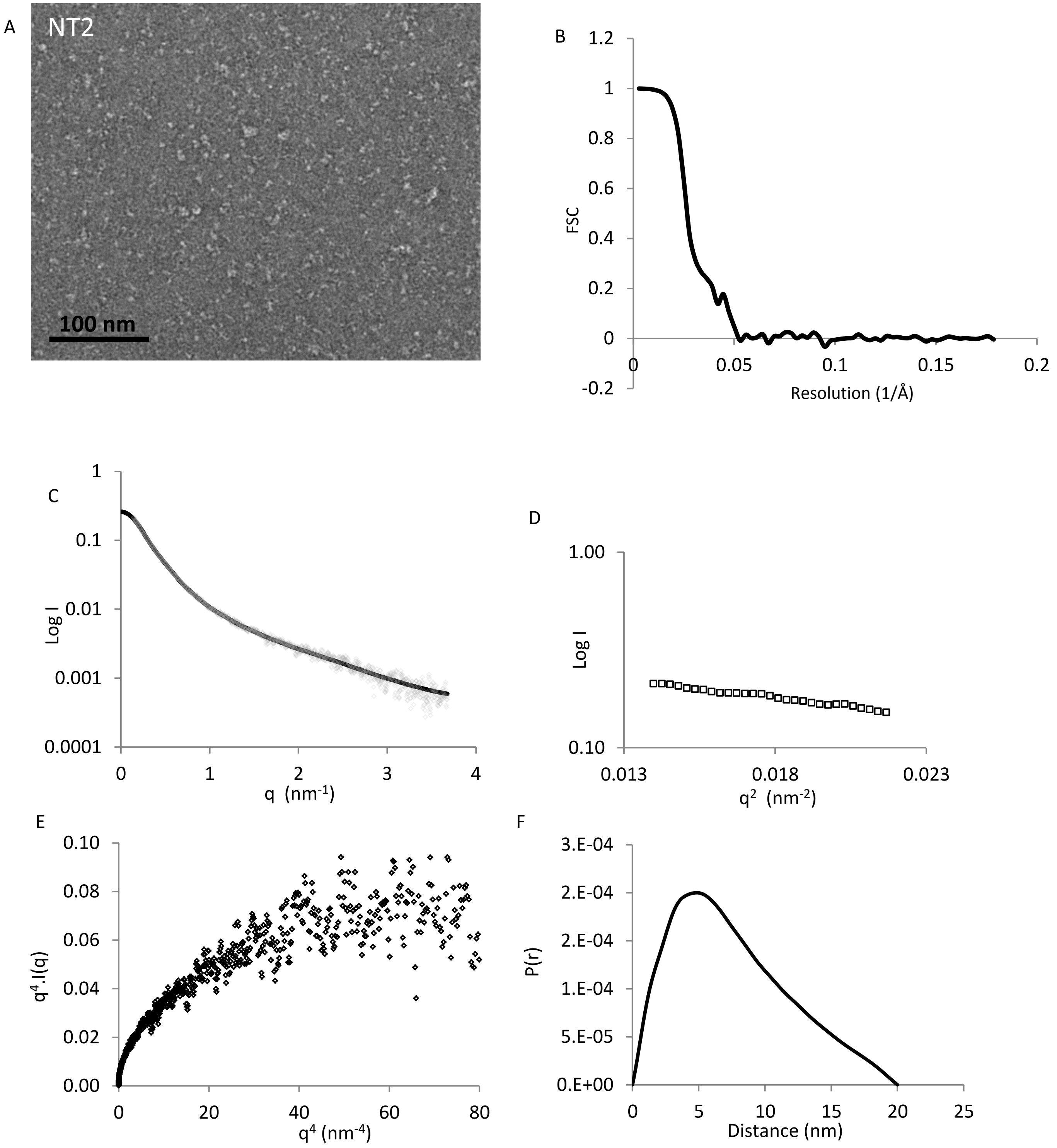

Supplementary Figure 2: NT2 electron microscopy and SAXS (A) Negatively stained EM image of the NT2 region in the presence of calcium. (B) Fourier shell correlation between even and odd particle models as a function of resolution. Resolution of the 3D model was determined as 24Å using Gold Standard 0.143 cutoff. (C) SEC-SAXS data (grey) compared to representative ab initio model fit (black line). (D) Guinier plot of the low q region gives rise to an Rg of 6nm. (E) Plateau in the Porod-Debye plot suggests overall structure is inflexible. (F) P(r) fit generated using Gnom showing a Dmax of 20nm.

Supplementary Figure 3

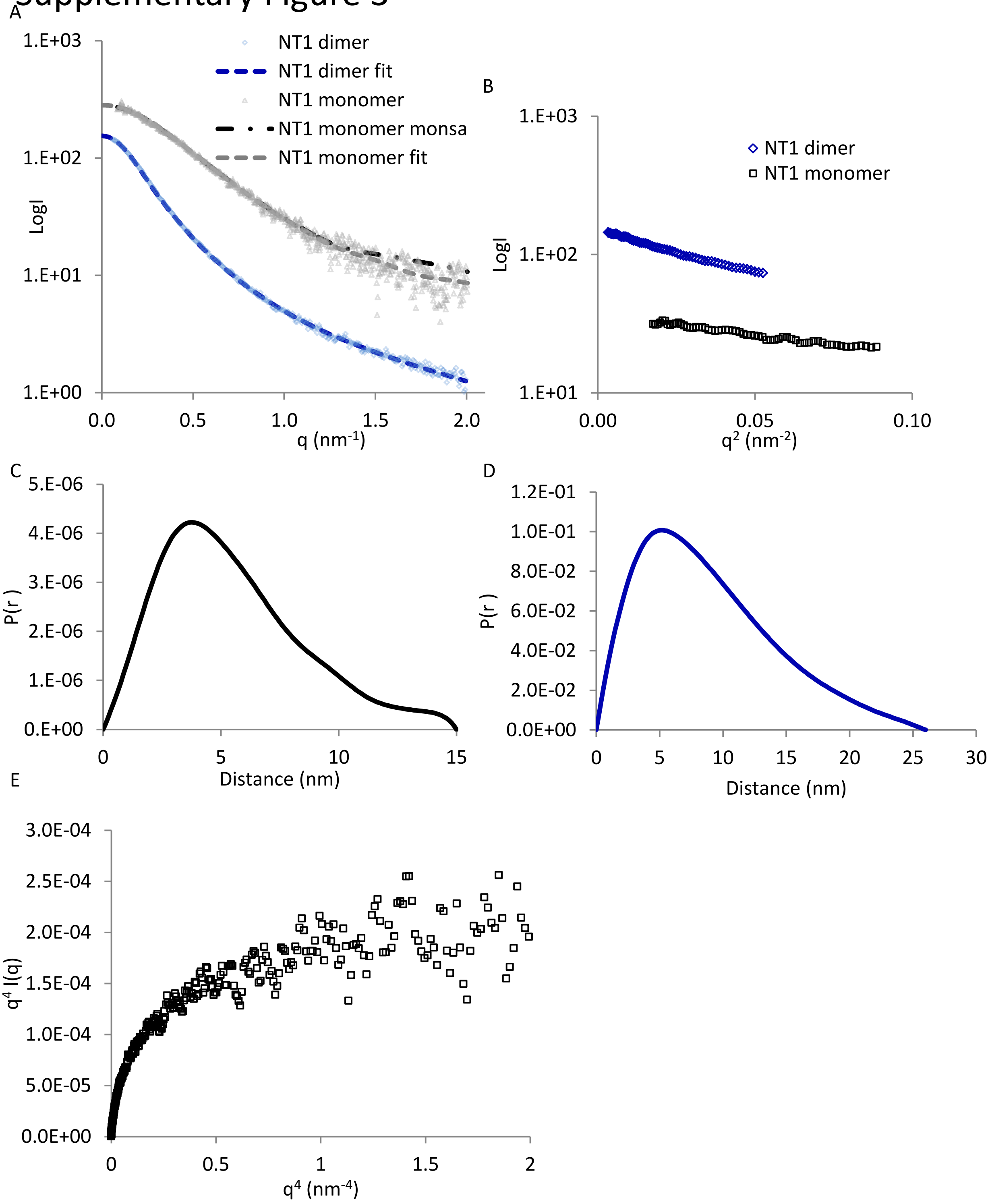

Supplementary Figure 3: SAXS analysis of monomeric and dimeric forms of NT1 (A) SEC-SAXS data for the monomer (black) compared to the Monsa fit and for the dimer (blue) compared with a representative ab initio model. (B) Guinier plots of the low q regions give rise to an Rg of 4.3 nm for the monomer and 6.6 nm for the dimer. (C) P(r) fit generated using Gnom of the monomeric form showing a Dmax of 15 nm. (D) P(r) fit generated using Gnom of the dimeric form showing a Dmax of 26 nm. (E) Porod-Debye (q^4) plot showing a plateau which suggests the overall structure is inflexible.

Supplementary Figure 4

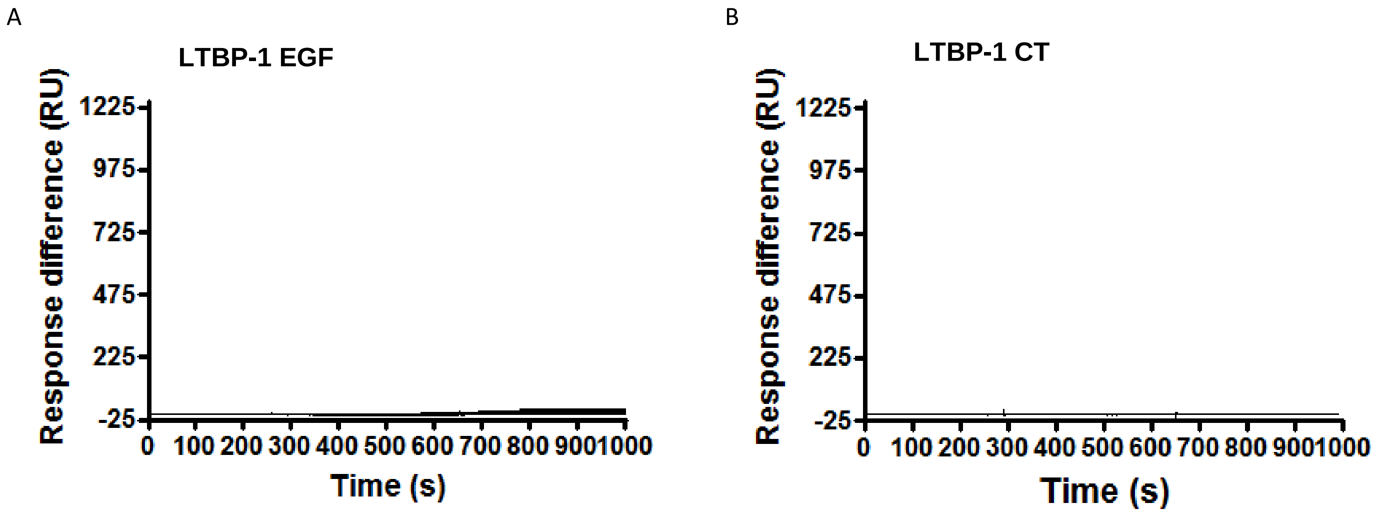

Supplementary Figure 4: Analysis of interaction between EGF and CT constructs and heparin: No binding was detected between immobilized heparin dp-20 and (A) the cbEGF construct and (B) the CT construct in the presence of calcium or EGTA using SPR.

Supplementary Figure 5

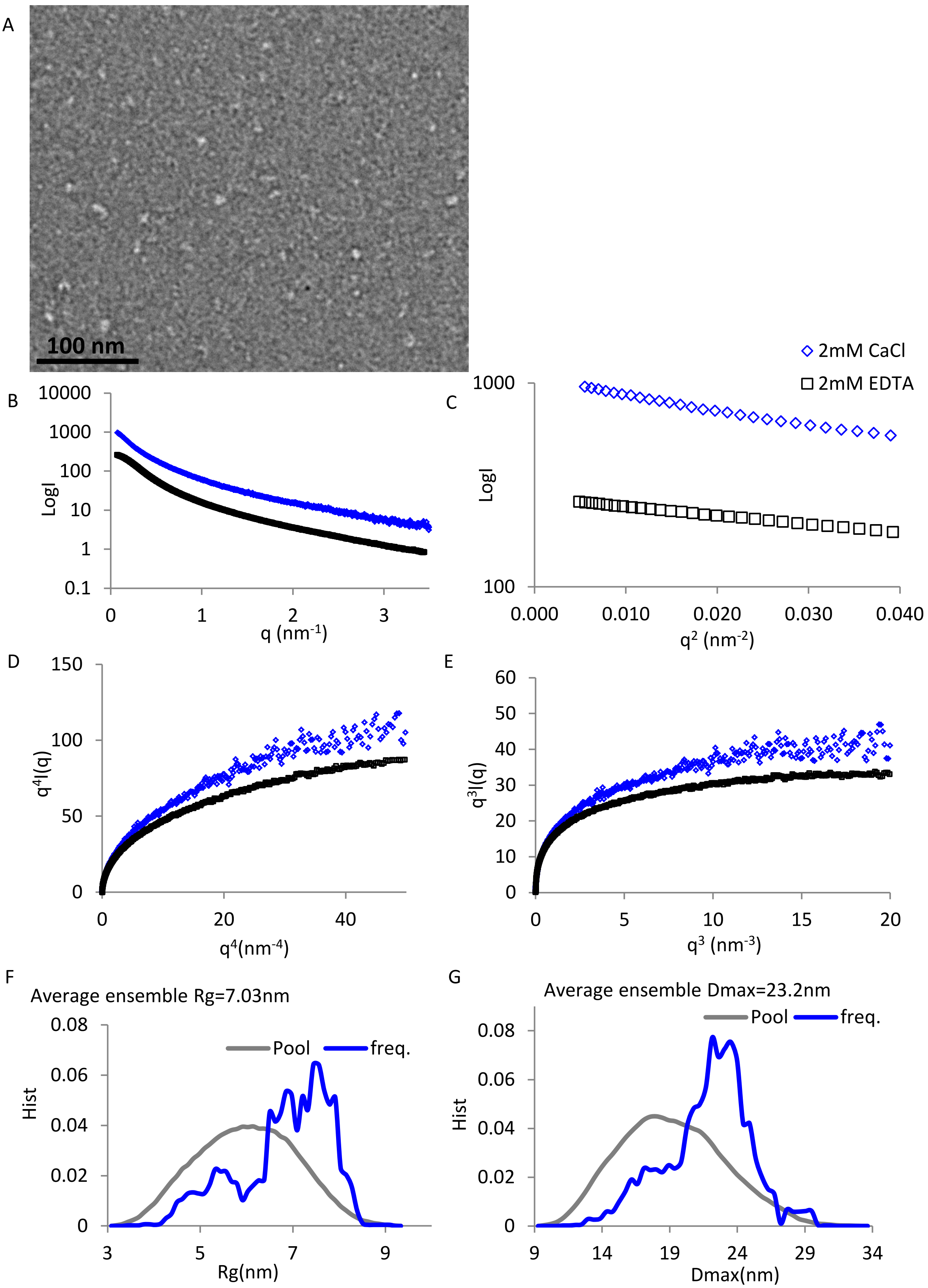

Supplementary Figure 5: Effect of calcium on cbEGF region structure (A) Negatively stained EM image of the cbEGF region. (B) SEC-SAXS profile of intensity as a function of the scattering angle (q) for the cbEGF region in the presence of either 2mM calcium chloride (blue) or 2mM EDTA (black). (C) Guinier plot showing a sharp decrease in Rg (7.6 nm to 5.4 nm) when calcium is removed. (D) Porod-Debye (q^4) and q^3 (E) plots. The power-law relationship between q and I for globular and flexible proteins suggests that in both calcium-containing and calcium-free conditions the cbEGF region is flexible. Ensemble Optimization Method (EOM) was performed for the cbEGF region in the presence of calcium. (F) Rg Distribution of pool of models generated by EOM (grey) and frequency of occurrence according to the fit (blue). Ensemble average = 7.03nm. (G) Dmax Distribution, ensemble average = 23.2nm.

Supplementary Figure 6

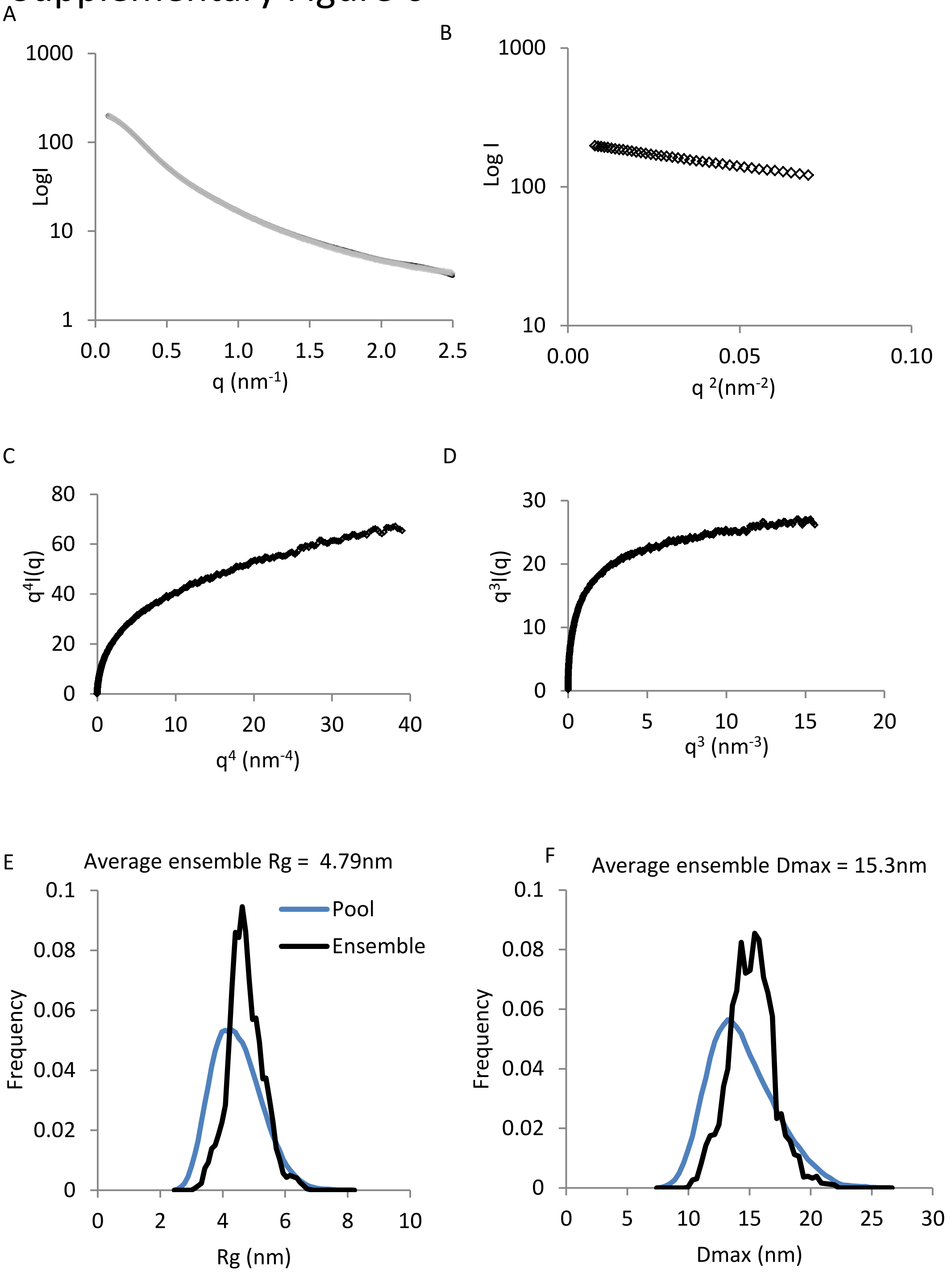

Supplementary Figure 6: The C-terminal LTBP1 region is flexible (A) SEC-SAXS profile log intensity as a function of q with the EOM ensemble model fit (black line; chi^2=1.20). (B) Guinier region showing Rg = 4.9nm. (C) Porod-Debye and q^3 (D) plots showing absence of a plateau at q^4 present at q^3 indicative of flexibility. (E) Rg distribution of the model pool (blue) and predicted frequency of these models occurring in the sample (black). (F) Dmax distribution from the model pool and predicted frequency based on SAXS data.

Supplementary Figure 7

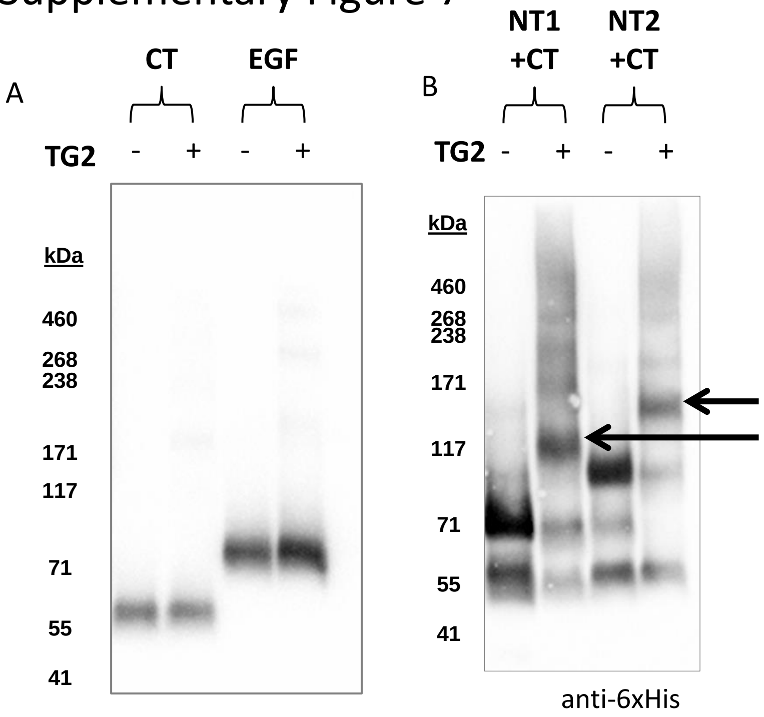

C

Band 1

```
   1 MDTKLMCLLF FFSLPPLLVS NHTGRIKVVF TPSICKVTCT KGSCQNSCEK GNTTTLISEN
  61 GHAADTLTAT NFRVVICHLP CMNGGQCSSR DKCQCPPNFT GKLCQIPVHG ASVPKLYQHS
 121 QQPGKALGTH VIHSTHTLPL TVTSQQGVKV KFPPNIVNIH VKHPPEASVQ IHQVSRIDGP
 181 TGQKTKEAQP GQSQVSYQGL PVQKTQTIHS TYSHQQVIPH VYPVAAKTQL GRCFQETIGS
 241 QCGKALPGLS KQEDCCGTVG TSWGFNKCQK CPKKPSYHGY NQMMECLPGY KRVNNTFCQD
 301 INECQLQGVC PNGECLNTMG SYRCTCKIGF GPDPTFSSCV PDPPVISEEK GPCYRLVSSG
 361 RQCMHPLSVH LTKQLCCCSV GKAWGPHCEK CPLPGTAAFK EICPGGMGYT VSGVHRRRPI
 421 HHHVGKGPVF VKPKNTQPVA KSTHPPPLPA KEEPVEALTF SREHGPGVAE PEVATAPPEK
 481 EIPSLDQEKT KLEPGQPQLS PGISTIHLHP QFPVVIEKTS PPVPVEVAPE ASTSSASQVI
 541 APTQVTEINE CTVNPDICGA GHCINLPVRY TCICYEGYRF SEQQRKCVDI DECTQVQHLC
 601 SQGRCENTEG SFLCICPAGF MASEEGTNCI DVDECLRPDV CGEGHCVNTV GAFRCEYCDS
 661 GYRMTQRGRC EDIDECLNPS TCPDEQCVNS PGSYQCVPCT EGFRGWNGQC LDVDECLEPN
 721 VCANGDCSNL EGSYMCSCHK GYTRTPDHKH CRDIDECQQG NLCVNGQCKN TEGSFRCTCG
 781 QGYQLSAAKD QCEDIDECQH RHLCAHGQCR NTEGSFQCVC DQGYRASGLG DHCEDINECL
 841 EDKSVCQRGD CINTAGSYDC TCPDGFQLDD NKTCQDINEC EHPGLCGPQG ECLNTEGSFH
 901 CVCQQGFSIS ADGRTCEDID ECVNNTVCDS HGFCDNTAGS FRCLCYQGFQ APQDGQGCVD
 961 VNECELLSGV CGEAFCENVE GSFLCVCADE NQEYSPMTGQ CRSRTSTDLD VDVDQPKEEK
1021 KECYYNLNDA SLCDNVLAPN VTKQECCCTS GAGWGDNCEI FPCPVLGTAE FTEMCPKGKG
1081 FVPAGESSSE AGGENYKDAD ECLLFGQEIC KNGFCLNTRP GYECYCKQGT YYDPVKLQCF
1141 DMDECQDPSS CIDGQCVNTE GSYNCFCTHP MVLDASEKRC IRPAESNEQI EETDVYQDLC
1201 WEHLSDEYVC SRPLVGKQTT YTECCCLYGE AWGMQCALCP LKDSDDYAQL CNIPVTGRRQ
1261 PYGRDALVDF SEQYTPEADP YFIQDRFLNS FEELQAEECG ILNGCENGRC VRVQEGYTCD
1321 CFDGYHLDTA KMTCVDVNEC DELNNRMSLC KNAKCINTDG SYKCLCLPGY VPSDKPNYCT
1381 PLNTALNLEK DSDLE
```

Band 2

```
   1 MDTKLMCLLF FFSLPPLLVS NHTGRIKVVF TPSICKVTCT KGSCQNSCEK GNTTTLISEN
  61 GHAADTLTAT NFRVVICHLP CMNGGQCSSR DKCQCPPNFT GKLCQIPVHG ASVPKLYQHS
 121 QQPGKALGTH VIHSTHTLPL TVTSQQGVKV KFPPNIVNIH VKHPPEASVQ IHQVSRIDGP
 181 TGQKTKEAQP GQSQVSYQGL PVQKTQTIHS TYSHQQVIPH VYPVAAKTQL GRCFQETIGS
 241 QCGKALPGLS KQEDCCGTVG TSWGFNKCQK CPKKPSYHGY NQMMECLPGY KRVNNTFCQD
 301 INECQLQGVC PNGECLNTMG SYRCTCKIGF GPDPTFSSCV PDPPVISEEK GPCYRLVSSG
 361 RQCMHPLSVH LTKQLCCCSV GKAWGPHCEK CPLPGTAAFK EICPGGMGYT VSGVHRRRPI
 421 HHHVGKGPVF VKPKNTQPVA KSTHPPPLPA KEEPVEALTF SREHGPGVAE PEVATAPPEK
 481 EIPSLDQEKT KLEPGQPQLS PGISTIHLHP QFPVVIEKTS PPVPVEVAPE ASTSSASQVI
 541 APTQVTEINE CTVNPDICGA GHCINLPVRY TCICYEGYRF SEQQRKCVDI DECTQVQHLC
 601 SQGRCENTEG SFLCICPAGF MASEEGTNCI DVDECLRPDV CGEGHCVNTV GAFRCEYCDS
 661 GYRMTQRGRC EDIDECLNPS TCPDEQCVNS PGSYQCVPCT EGFRGWNGQC LDVDECLEPN
 721 VCANGDCSNL EGSYMCSCHK GYTRTPDHKH CRDIDECQQG NLCVNGQCKN TEGSFRCTCG
 781 QGYQLSAAKD QCEDIDECQH RHLCAHGQCR NTEGSFQCVC DQGYRASGLG DHCEDINECL
 841 EDKSVCQRGD CINTAGSYDC TCPDGFQLDD NKTCQDINEC EHPGLCGPQG ECLNTEGSFH
 901 CVCQQGFSIS ADGRTCEDID ECVNNTVCDS HGFCDNTAGS FRCLCYQGFQ APQDGQGCVD
 961 VNECELLSGV CGEAFCENVE GSFLCVCADE NQEYSPMTGQ CRSRTSTDLD VDVDQPKEEK
1021 KECYYNLNDA SLCDNVLAPN VTKQECCCTS GAGWGDNCEI FPCPVLGTAE FTEMCPKGKG
1081 FVPAGESSSE AGGENYKDAD ECLLFGQEIC KNGFCLNTRP GYECYCKQGT YYDPVKLQCF
1141 DMDECQDPSS CIDGQCVNTE GSYNCFCTHP MVLDASEKRC IRPAESNEQI EETDVYQDLC
1201 WEHLSDEYVC SRPLVGKQTT YTECCCLYGE AWGMQCALCP LKDSDDYAQL CNIPVTGRRQ
1261 PYGRDALVDF SEQYTPEADP YFIQDRFLNS FEELQAEECG ILNGCENGRC VRVQEGYTCD
1321 CFDGYHLDTA KMTCVDVNEC DELNNRMSLC KNAKCINTDG SYKCLCLPGY VPSDKPNYCT
1381 PLNTALNLEK DSDLE
```

Band 3

```
   1 MDTKLMCLLF FFSLPPLLVS NHTGRIKVVF TPSICKVTCT KGSCQNSCEK GNTTTLISEN
  61 GHAADTLTAT NFRVVICHLP CMNGGQCSSR DKCQCPPNFT GKLCQIPVHG ASVPKLYQHS
 121 QQPGKALGTH VIHSTHTLPL TVTSQQGVKV KFPPNIVNIH VKHPPEASVQ IHQVSRIDGP
 181 TGQKTKEAQP GQSQVSYQGL PVQKTQTIHS TYSHQQVIPH VYPVAAKTQL GRCFQETIGS
 241 QCGKALPGLS KQEDCCGTVG TSWGFNKCQK CPKKPSYHGY NQMMECLPGY KRVNNTFCQD
 301 INECQLQGVC PNGECLNTMG SYRCTCKIGF GPDPTFSSCV PDPPVISEEK GPCYRLVSSG
 361 RQCMHPLSVH LTKQLCCCSV GKAWGPHCEK CPLPGTAAFK EICPGGMGYT VSGVHRRRPI
 421 HHHVGKGPVF VKPKNTQPVA KSTHPPPLPA KEEPVEALTF SREHGPGVAE PEVATAPPEK
 481 EIPSLDQEKT KLEPGQPQLS PGISTIHLHP QFPVVIEKTS PPVPVEVAPE ASTSSASQVI
 541 APTQVTEINE CTVNPDICGA GHCINLPVRY TCICYEGYRF SEQQRKCVDI DECTQVQHLC
 601 SQGRCENTEG SFLCICPAGF MASEEGTNCI DVDECLRPDV CGEGHCVNTV GAFRCEYCDS
 661 GYRMTQRGRC EDIDECLNPS TCPDEQCVNS PGSYQCVPCT EGFRGWNGQC LDVDECLEPN
 721 VCANGDCSNL EGSYMCSCHK GYTRTPDHKH CRDIDECQQG NLCVNGQCKN TEGSFRCTCG
 781 QGYQLSAAKD QCEDIDECQH RHLCAHGQCR NTEGSFQCVC DQGYRASGLG DHCEDINECL
 841 EDKSVCQRGD CINTAGSYDC TCPDGFQLDD NKTCQDINEC EHPGLCGPQG ECLNTEGSFH
 901 CVCQQGFSIS ADGRTCEDID ECVNNTVCDS HGFCDNTAGS FRCLCYQGFQ APQDGQGCVD
 961 VNECELLSGV CGEAFCENVE GSFLCVCADE NQEYSPMTGQ CRSRTSTDLD VDVDQPKEEK
1021 KECYYNLNDA SLCDNVLAPN VTKQECCCTS GAGWGDNCEI FPCPVLGTAE FTEMCPKGKG
1081 FVPAGESSSE AGGENYKDAD ECLLFGQEIC KNGFCLNTRP GYECYCKQGT YYDPVKLQCF
1141 DMDECQDPSS CIDGQCVNTE GSYNCFCTHP MVLDASEKRC IRPAESNEQI EETDVYQDLC
1201 WEHLSDEYVC SRPLVGKQTT YTECCCLYGE AWGMQCALCP LKDSDDYAQL CNIPVTGRRQ
1261 PYGRDALVDF SEQYTPEADP YFIQDRFLNS FEELQAEECG ILNGCENGRC VRVQEGYTCD
1321 CFDGYHLDTA KMTCVDVNEC DELNNRMSLC KNAKCINTDG SYKCLCLPGY VPSDKPNYCT
1381 PLNTALNLEK DSDLE
```

Supplementary Figure 7: TG2-mediated cross-linking of LTBP-1 N- and C-termini (A) Western blot under reducing conditions using anti-6xHistidine primary antibody for CT and EGF alone in the presence and absence of TG2. (B) NT1 and NT2 incubated with CT in the presence and absence of TG2 showing additional bands corresponding to crosslinked N- and C-terminal LTBP-1 when the N- and C-terminal constructs are incubated together in the presence of TG2 (arrows). (C) MS peptides from bands shown in Figure 5C. Band 1 (NT1 and CT constructs incubated with TG2) contains peptides from both constructs, whereas band 2 contains only C-terminal peptides and band 3 N-terminal peptides only.
